# Technical issues and conservation conditions of medicines in the primary health care of the Brazilian Unified Health System

**DOI:** 10.11606/S1518-8787.2017051007106

**Published:** 2017-09-22

**Authors:** Ediná Alves Costa, Patrícia Sodré Araújo, Marcelo Tavares Pereira, Ana Cristina Souto, Gisélia Santana Souza, Augusto Afonso Guerra, Francisco de Assis Acurcio, Ione Aquemi Guibu, Juliana Alvares, Karen Sarmento Costa, Margô Gomes de Oliveira Karnikowski, Orlando Mario Soeiro, Silvana Nair Leite

**Affiliations:** I Instituto de Saúde Coletiva. Universidade Federal da Bahia. Salvador, BA, Brasil; II Departamento de Ciências da Vida. Universidade do Estado da Bahia. Salvador, BA, Brasil; III Faculdade de Farmácia. Universidade Federal da Bahia. Salvador, BA, Brasil; IV Departamento de Farmácia Social. Faculdade de Farmácia. Universidade Federal de Minas Gerais. Belo Horizonte, MG, Brasil; V Departamento de Saúde Coletiva. Faculdade de Ciências Médicas. Santa Casa de São Paulo. São Paulo, SP, Brasil; VI Núcleo de Estudos de Políticas Públicas. Universidade Estadual de Campinas. Campinas, SP, Brasil; VII Programa de Pós-Graduação em Saúde Coletiva. Departamento de Saúde Coletiva. Faculdade de Ciências Médicas. Universidade Estadual de Campinas. Campinas, SP, Brasil; VIII Programa de Pós-Graduação em Epidemiologia. Faculdade de Medicina. Universidade Federal do Rio Grande do Sul. Porto Alegre, RS, Brasil; IX Faculdade de Ceilândia. Universidade de Brasília. Brasília, DF, Brasil; X Faculdade de Ciências Farmacêuticas. Pontifícia Universidade Católica de Campinas. Campinas, SP, Brasil; XI Departamento de Ciências Farmacêuticas. Universidade Federal de Santa Catarina. Florianópolis, SC, Brasil

**Keywords:** Drugs for Primary Health Care, Drug Storage, Pharmaceutical Services, Pharmacovigilance, Unified Health System, Health Surveillance, Medicamentos para Atenção Básica, Armazenamento de Medicamentos, Assistência Farmacêutica, Farmacovigilância, Sistema Único de Saúde, Vigilância Sanitária

## Abstract

**OBJECTIVE:**

To characterize the technical issues and conditions of medicines conservation in Primary Health Care of Brazilian regions, responsible for pharmacy/dispensing unit profile; environmental, storage, and dose fractioning conditions; inventory control and waste management; fire and electrical failure safety items; transportation problems; advertising regulation; and pharmacovigilance.

**METHODS:**

This article is part of the *Pesquisa Nacional sobre Acesso, Utilização e Promoção do Uso Racional de Medicamentos – Serviços* (National Survey on Access, Use and Promotion of Rational Use of Medicines – Services)–, a cross-sectional and exploratory study, of evaluative nature, consisting of an information survey within a representative sample of municipalities, stratified by Brazilian regions, which constitute the study domains, and a sample of Primary Health Care services. Pharmaceutical services (PS) were directly observed with photographic record and face-to-face interviews with those responsible for the dispensing of medicines and over the telephone with those responsible for pharmaceutical services. Data were processed with the SPSS^®^ software version 21.

**RESULTS:**

The investigated dimensions showed relevant deficiencies and inequalities between the regions, generally more favorable in the Southeast and Midwest regions and weaker in the Northeast and North regions. We verified non-compliance with technical requirements and conditions essential to the conservation of medicines, which may interfere with the maintenance of stability and, thus, on their quality, efficacy, and safety. The regulation of advertising/promotion of medicines is still incipient and there is some progress in the structuring of mechanisms regarding pharmacovigilance.

**CONCLUSIONS:**

The sanitary situation of medicines in Brazilian Primary Health Care is alarming due to the violation of the specific sanitary legislation for dispensing establishments and due to a wide range of requirements essential to the conservation of medicines. We observed a disconnection between the efforts made in the Brazilian Unified Health System to promote access to medicines for all population and the organization and qualification of pharmaceutical services.

## RESUMO

**OBJETIVO:**

Caracterizar a situação sanitária dos medicamentos na Atenção Básica, nas regiões brasileiras, quanto a requisitos técnico-sanitários, responsável pela farmácia/unidade de dispensação, condições ambientais, de armazenamento, e de fracionamento, controle de estoque e gerenciamento de resíduos, itens de segurança contra incêndio e pane elétrica, problemas no transporte, regulamentação da propaganda e farmacovigilância.

**MÉTODOS:**

Artigo integrante da Pesquisa Nacional sobre Acesso, Utilização e Promoção do Uso Racional de Medicamentos – Serviços, um estudo transversal, exploratório, de natureza avaliativa, composto por um levantamento de informações numa amostra representativa de municípios, estratificada pelas regiões brasileiras, que constituem domínios do Estudo, e uma amostra de serviços de Atenção Básica. Realizou-se observação direta dos serviços farmacêuticos com registro fotográfico e entrevistas presenciais com os responsáveis pela entrega de medicamentos e por telefone com o responsável pela assistência farmacêutica. Os dados foram processados com o *software* SPSS® versão 21.

**RESULTADOS:**

As dimensões investigadas mostraram deficiências relevantes e desigualdades entre as regiões, em geral mais favoráveis nas regiões Sudeste e Centro-Oeste e mais deficitárias nas regiões Nordeste e Norte. Constatou-se descumprimento de requisitos técnicos e sanitários imprescindíveis à conservação dos medicamentos que podem interferir na manutenção da estabilidade e, assim, na sua qualidade, eficácia e segurança. A regulação da propaganda/promoção de medicamentos ainda é incipiente e existe algum avanço na estruturação de mecanismos em relação à farmacovigilância.

**CONCLUSÕES:**

A situação sanitária dos medicamentos na Atenção Básica no Brasil desperta preocupações pelo descumprimento da legislação sanitária específica para os estabelecimentos de dispensação e de um amplo conjunto de requisitos imprescindíveis à conservação dos medicamentos. Constatou-se um descompasso entre os esforços no âmbito do Sistema Único de Saúde para promover o acesso aos medicamentos para toda a população e a organização e qualificação dos serviços farmacêuticos.

## INTRODUCTION

Medicines have a hybrid nature, present benefits, but also health risks; they are health care inputs and, at the same time, a commodity on the market that requires, therefore, sanitary regulation in all stages of their production cycle. Thus, the State is responsible for regulating the production, trade, transport, storage, dispensing, and use of medicines so they can achieve their purpose in the health system^6^. Furthermore, it is up to the State to regulate the management and final disposal of waste to protect the health of workers and population, as well as the environment.

The history of medicines in Public Health has been marked by successes and tragedies, which encouraged national states to implement strong regulatory systems, in legislation and institutional structures, as well as the formulation and implementation of concepts that mean safety assurance measures before the medicine is delivered to consumption^15^. The regulatory systems must also act to protect the population’s health from ineffective and unnecessary medicines and promote the rational use, in addition to intervene in market strategies that aim to stimulate the consumption of medicines^5^ as common consumer goods.

The core of the sanitary regulation of medicines focuses on quality, safety, efficiency, and information; it covers the formulation of norms and standards, the product registration process since the authorization of clinical trials, the licensing of facilities and personnel, the quality control of the drugs and the monitoring of adverse reactions, the emission of alerts and product recall. The information reports from the analysis and approval of reports and package inserts of the product to the advertising and promotion of the medicine^27^.

Serious mistakes in medicine manufacturing propelled the World Health Organization (WHO) to establish good manufacturing practices, which became mandatory for the pharmaceutical industry. The concept of good practices was incorporated to the various activities related to medicine, such as distribution, transportation and storage, marketing, dispensing, manipulation of magistral and officinal preparations, because any improper activity can affect the quality.

The sanitary control of medicines represents a challenge in the performance of regulatory systems around the world. Poor quality, counterfeit medicines and other irregularities are very common in the consumer market and any class of medicines can be affected. It is difficult to estimate the amount in circulation on the market and the impact of this problem on society, for the deaths and illnesses caused, for the time and financial resources with their use^10^.

Sanitary regulations, promotion of rational use, and guarantee of safety, efficacy, and quality of medicines are guidelines of the *Política Nacional de Medicamentos* (PNM – National Drug Policy), adopted by the Brazilian Ministry of Health (MS) in 1998^a^. The *Sistema Nacional de Vigilância Sanitária* (SNVS – Brazilian Health Surveillance System) – subsystem of the Brazilian Unified Health System (SUS) – triggers regulatory and control technologies in the entire production cycle of these goods, with competencies shared by the three levels of government. Despite the advances in sanitary regulation, with the creation of the *Agência Nacional de Vigilância Sanitária* (Anvisa – National Sanitary Surveillance Agency) in 1999, SNVS is still deficient in monitoring the compliance with the norms, especially in public services^7^, in which the political power of health surveillance is even more limited.

The issue of pharmaceuticals waste and their final disposal, as part of health care waste, is the object of regulation by Anvisa/MS and by the Ministry of Environment, which established standards for the management of waste generated in health establishments – RDC no. 306/2004 of Anvisa^b^ and Resolution no. 358/2005 of the *Conselho Nacional do Meio Ambiente* (Conama – National Environment Council)^c^.

In addition to the health surveillance regulations, the Brazilian Ministry of Health released guidelines for SUS pharmacies, aiming to guide the conception, structuring, and elaboration of the Handbook of Pharmaceutical Practices. There are references to the sanitary and technical requirements for the operation of the pharmaceutical establishment^17^ ^,^ ^18^.

The sanitary situation of medicines in Brazil has not been an object of extensive research yet. Thus, this study aimed to characterize the sanitary situation of medicines in Primary Health Care, in the different regions of the Country, regarding sanitary-technical documentation and responsible for pharmacy/dispensing unit, dose fractioning and storage conditions, environmental conditions within the storage and dispensing areas, inventory control, waste management, advertising regulations, and initiatives concerning pharmacovigilance.

## METHODS

This article is part of the *Pesquisa Nacional sobre Acesso, Utilização e Promoção do Uso Racional de Medicamentos – Serviços, 2015* (PNAUM – National Survey on Access, Use and Promotion of Rational Use of Medicines – Services, 2015), which aimed to characterize the organization of pharmaceutical services in the Primary Health Care of SUS, aiming at the access and promotion of rational use of medicines, as well as to identify and discuss factors that interfere in the consolidation of pharmaceutical services in the cities.

PNAUM is a cross-sectional, exploratory study, of evaluative nature, consisting of an information survey in a sample of Primary Health Care services, in representative cities of the regions of Brazil, with direct observation of pharmaceutical services and interviews. Several study populations were considered in the sampling plan, according to the methodological details presented in another article^2^, with samples stratified by regions, which constitute the study domains.

The interviews were carried out with specific questionnaires for each category of respondent: face-to-face with those responsible for the dispensing of medicines, doctors, and patients and by phone with those responsible for pharmaceutical services and the municipal secretaries of health. During the observation of pharmacies/medicine dispensing units and locations of storage and delivery of medicines, we used a script with photographic record, to verify the technical-sanitary documentation, the medicine storage and delivery conditions, the record of the activities, availability of the selected medicines, the existence of expired medicines, and location for storage of those unfit for use.

The interviews and observations were performed by trained interviewers. The observer was accompanied by a professional of the health personnel who knew the locations of observation in the unit or in another place of reference. The items of the script were filled based on the observation and information of the professional who accompanied the interviewer.

The sanitary conditions of the medicines were based on observation data of pharmacies/dispensing units of the services’ sample. Data on transportation problems were obtained from the interviews with coordinators of pharmaceutical services, whereas data related to the control of advertising of medicines, pharmacovigilance, inventory control, and waste management were gathered from the interviews with the pharmacists responsible for the dispensing of medicines. Data were analyzed using SPSS^®^ software, version 21, in the analysis module for complex samples. For analysis of statistical association, we applied the Chi-square test with a significance level of p < 0.05.

PNAUM – Services was approved by the National Research Ethics Committee (Opinion 398,131/2013), with clarification of the research’s objectives to respondents and signing of the informed consent form.

## RESULTS

We analyzed 1,175 pharmacies/dispensing units (86.3% of the estimated sample), 495 pharmaceutical services coordinators (84.5% of the estimated sample), and 1,139 professionals responsible for the dispensing of medicines (83.6% of the estimated sample). Of these, 32.7% were pharmacists.

Table 1 shows the sanitary conditions of pharmacies/medicine dispensing units in the regions. Differences were observed between the regions in all dimensions. In general, the Northeast region presented poorer conditions, followed by the North, while the Southeast presented more positive results, followed by the Midwest.

**Table 1:** Sanitary conditions in pharmacies/medicine dispensing units in the Primary Health Care, according to the regions of Brazil. National Survey on Access, Use and Promotion of Rational Use of Medicines – Services, 2015. (n = 1,175)

Dimension/Variable	North	Northeast	Midwest	Southeast	South	Brazil
					
	% (95%CI)	% (95%CI)	% (95%CI)	% (95%CI)	% (95%CI)	% (95%CI)
Technical-sanitary documentation*						
Location and Operation License	49.1 (47.2–51.0)	18.2 (17.3–19.1)	77.0 (75.7–8.2)	72.5 (71.3–73.6)	51.8 (50.8–52.8)	46.6 (46.0–47.2)
Sanitary License (License visible and current)	25.8 (24.3–27.3)	24.7 (23.9–25.6)	58.8 (57.8–59.8)	57.6 (56.6–58.5)	44.8 (43.7–45.8)	40.4 (39.8–40.9)
Fire Department License	37.9 (36.1–39.7)	6.6 (6.0–7.3)	41.6 (40.4–42.8)	48.3 (47.1–49.5)	40.0 (38.9–41.0)	29.7 (29.2–30.3)
Certificate of Technical Responsibility	20.0 (18.6–21.4)	15.3 (14.6–16.1)	63.4 (62.4–64.4)	57.9 (56.8–58.9)	53.6 (52.5–54.6)	38.1 (37.7–38.6)
Professional responsible for Pharmacy/Dispensing Unit						
Pharmacist*	26.8 (24.8–28.8)	18.5 (17.7–19.4)	66.9 (65.6–68.3)	72.0 (71.0–72.9)	44.8 (43.8–45.8)	43.0 (42.5–43.5)
Another higher-level health professional	12.5 (11.2–14.0)	19.1 (18.3–20.0)	16.9 (15.7–18.0)	15.0 (14.4–15.7)	22.4 (21.7–23.2)	17.8 (17.4–18.2)
Storage area conditions*						
Air conditioner	59.0 (57.3–60.8)	21.3 (20.4–22.2)	72.7 (71.5–73.8)	46.0 (44.8–47.2)	37.1 (36.0–38.1)	37.7 (37.1–38.2)
Cabinet with key for controlled medicines in the dispensing units	38.2 (36.2–40.2)	22.6 (21.7–23.6)	65.1 (64.0–66.3)	63.4 (62.3–64.4)	48.9 (48.0–49.9)	43.4 (42.9–44.0)
Freezer/Refrigerator for unique storage of medicines	37.2 (35.2–39.1)	21.3 (20.3–22.3)	50.7 (49.4–52.0)	76.0 (75.2–76.9)	56.8 (56.1–57.6)	47.2 (46.7–47.7)
Medicines in direct contact with the floor or walls	22.2 (20.7–23.9)	11.6 (10.9–12.3)	8.0 (7.1–9.0)	11.2 (10.5–11.9)	21.6 (20.8–22.4)	13.9 (13.4–14.2)
Control of entry and transit of people	49.5 (47.6–51.3)	63.8 (62.8–64.7)	58.8 (57.5–60.1)	64.4 (63.4–65.3)	81.0 (80.1–81.9)	65.4 (64.9–65.9)
Cabinets or shelves for storage of products (medicines, inputs)	84.4 (82.9–85.8)	86.4 (85.5–87.1)	97.6 (97.2–98.0)	93.1 (92.7–93.4)	92.9 (92.2–93.5)	90.0 (89.7–90.4)
Pallets/platforms	13.8 (12.4–15.3)	13.8 (13.0–14.7)	30.8 (29.6–31.9)	48.8 (47.8–49.9)	30.1 (29.2–31.1)	28.4 (27.9–28.9)
Digital thermometer (room temperature)	11.3 (9.9–12.9)	6.1 (5.3–7.0)	39.5 (38.3–40.7)	51.6 (50.5–52.8)	28.9 (27.9–30.0)	26.4 (25.9–27.0)
Hygrometer (humidity)	3.2 (2.7–3.8)	2.1 (1.7–2.6)	21.1 (20.5–21.7)	17.8 (16.6–19.2)	14.7 (13.7–15.7)	10.3 (9.8–10.8)
Thermometer for refrigerator	25.0 (23.2–26.8)	16.2 (15.3–17.1)	43.5 (42.1–44.8)	71.4 (70.5–72.3)	50.2 (49.4–51.1)	41.3 (40.8–41.8)
Bin-type boxes for storage of medicines	12.5 (11.5–13.6)	9.2 (8.2–10.2)	25.5 (24.1–27.0)	56.6 (55.4–57.8)	36.4 (35.4–37.4)	29.7 (29.1–30.2)
Existence of at least one expired medicine in the inventory	23.1 (18.1–28.9)	42.2 (34.3–50.6)	44.2 (31.3–57.8)	32.2 (20.1–47.2)	21.8 (15.4–29.9)	34.2 (28.9–40.0)
Fire and electrical failure safety items*						
Fire prevention equipment	15.8 (14.4–17.2)	17.5 (16.8–18.2)	36.7 (35.6–37.9)	47.0 (45.8–48.2)	47.1 (46–48.1)	32.6 (32.1–33.1)
Electric power generator	4.1 (3.5–4.8)	1.0 (0.9–1.2)	5.8 (5.7–5.8)	3.7 (2.8–5.0)	2.9 (2.8–3.0)	2.7 (2.4–3.1)

* p < 0.05

Source: PNAUM – Services, 2015.

In the dimension of technical-sanitary documentation, pharmacies/dispensing units were deficient in all the items, with statistically significant differences between the regions. The lowest percentage of units with sanitary license (24.7%) was found in the Northeast, and the highest (58.8%) was found in the Midwest. These same regions also showed the lowest (15.3%) and highest percentage (63.4%), respectively, of the technical responsibility certificate.

Regarding the responsibility for the pharmacy/medicine dispensing unit, in Brazil, only 43% of these services rely on a responsible pharmacist. The differences between the regions ranged from 18.5%, in the Northeast, to 72%, in the Southeast, and they were significant. In conflict with a technical necessity and the sanitary legislation, this function is also performed by other health professionals and other workers – technician/nursing assistant or pharmacist assistant, community officer and even administrative officials and of general services – whose percentage was close to that of pharmacists.

The conditions of the storage area varied in all items between the regions, with statistically significant differences, showing, generally, to be poorer in the Northeast and North regions. In all dispensing units, at least one expired medicine was found among the 37 tracers; the highest percentages were in the Northeast (42.2%) and Midwest (44.2%). The medicines under special control, those for HIV/AIDS, and the herbal ones were the most frequent.

Fire and electrical failure safety items proved to be quite deficient and also varied between the regions, with statistically significant differences. Only 32.6% of the units in Brazil had fire prevention equipment and 2.7%, electric power generator.

Table 2 presents the environmental conditions of pharmacies/dispensing units, showing inequalities between the regions, generally more favorable in the Southeast and Midwest. In all items, there were deficiencies that can generate problems and affect the quality of the medicines. In Brazil, e.g., only 25.8% of these units control temperature; in the Northeast, the percentage is negligible (5.4%). In humidity control, the percentage in Brazil reaches only 11.9%.

**Table 2:** Environmental and dose fractioning conditions in pharmacies/medicine dispensing units in the Primary Health Care, according to the regions of Brazil. National Survey on Access, Use and Promotion of Rational Use of Medicines – Services, 2015. (n = 1,175)

Dimension/Variable	North	Northeast	Midwest	Southeast	South	Brazil
					
	% (95%CI)	% (95%CI)	% (95%CI)	% (95%CI)	% (95%CI)	% (95%CI)
Environmental conditions of pharmacy/dispensing unit						
^a^Has temperature control*	17.3 (15.6–19.2)	5.4 (4.7–6.3)	53.3 (52.0–54.5)	47.5 (46.3–48.6)	26.7 (25.7–27.8)	25.8 (25.3–26.3)
^a^Has air circulation system*	36.1 (34.1–38.1)	24.2 (23.3–25.2)	59.4 (58.2–60.6)	25.6 (24.3–26.9)	44.3 (43.3–45.2)	31.1 (30.5–31.6)
^a^Has humidity control*	12.1 (10.8–13.6)	2.4 (1.9–2.9)	27.2 (26.5–28.0)	19.1 (17.8–20.5)	14.6 (13.6–15.7)	11.9 (11.4–12.4)
^a^Specific area for the storage of medicines unfit for use*	32.4 (30.8–34.1)	15.0 (14.2–16.0)	54.8 (53.5–56.1)	63.2 (62.1–64.4)	45.9 (44.9–46.8)	39.9 (38.3–39.4)
^a^Temperature at the time of observation:*						
^a^Until 25°C	43.7 (41.9–45.5)	12.4 (11.6–13.3)	55.1 (53.9–56.3)	47.2 (46.1–48.4)	45.8 (44.8–46.8)	33.9 (33.3–34.4)
^b^Between 25° and 30°C	22.7 (21.3–24.1)	10.2 (9.4–11.1)	20.6 (19.2–21.9)	22.0 (21.2–22.9)	18.4 (17.9–18.9)	16.8 (16.4–17.3)
^b^Above 30°C	4.0 (3.1–5.1)	5.8 (5.5–6.0)	4.4 (3.8–5.0)	1.5 (1.2–1.8)	1.8 (1.5–2.2)	3.5 (3.4–3.7)
^b^Does not have thermometer/Temp. could not be verified.	29.6 (28.4–30.9)	71.7 (70.6–72.7)	19.9 (19.0–20.9)	29.3 (28.3–30.2)	34.0 (33.1–34.9)	45.8 (45.2–46.3)
^b^Allows incidence of direct sunlight on medicines	11.9 (11.0–12.9)	9.1 (8.7–9.5)	3.9 (3.3–4.5)	6.8 (6.1–7.5)	4.3 (3.8–5.0)	7.5 (7.2–7.8)
^b^Signs of the presence of rodents and insects*	9.2 (7.7–10.9)	1.9 (1.7–2.3)	6.6 (5.9–7.5)	2.5 (2.1–3.0)	8.6 (7.8–9.3)	4.1 (3.9–4.4)
^b^Presence of mold or infiltrations	19.4 (17.8–21.2)	13.2 (12.3–14.0)	22.4 (21.1–23.8)	8.8 (8.3–9.3)	24.0 (23.3–24.8)	14.7 (14.3–15.1)
Environmental conditions of dispensing area						
^a^Has temperature control*	19.8 (18.0–21.8)	13.7 (12.9–14.5)	62.8 (61.6–64.0)	47.0 (45.9–48.2)	35.6 (34.5–36.6)	31.2 (30.7–31.8)
^a^Has internal air circulation system*	38.9 (36.9–40.9)	26.2 (25.2–27.2)	60.9 (59.7–62.1)	32.0 (30.8–33.3)	45.9 (44.8–46.9)	34.4 (33.8–35.0)
^a^Has humidity control*	14.8 (13.5–16.3)	8.6 (8.0–9.3)	30.7 (30.0–31.5)	20.3 (19.0–21.6)	19.4 (18.4–20.5)	15.9 (15.4–16.4)
^b^Allows incidence of direct sunlight on medicines	14.1 (13.3–15.0)	10.3 (9.7–10.9)	3.9 (3.3–4.5)	7.5 (6.7–8.3)	6.5 (5.8–7.2)	8.7 (8.3–9.1)
^b^Signs of the presence of rodents and insects*	8.9 (7.3–10.7)	3.5 (3.0–4.1)	6.8 (6.0–7.7)	2.2 (1.8–2.8)	10.7 (9.9–11.6)	5.0 (4.6–5.3)
^b^Presence of mold or infiltrations*	22.8 (21.2–24.5)	14.8 (13.8–15.8)	19.9 (18,8–21.2)	9,8 (9.2–10.4)	25.5 (24.7–26.3)	16.0 (15.5–16.4)
Conditions of dose fractioning of medicines*						
Specific area for dose fractioning	10.6 (9.8–11.3)	3.2 (2.8–3.6)	25.9 (24.2–27.7)	31.3 (30.1–32.4)	3.8 (3.5–4.1)	12.8 (12.5–13.2)
Countertop covered with smooth and resistant material	7.8 (6.3–9.6)	7.3 (6.6–8.0)	35.8 (34.0–37.7)	41.2 (40.0–42.4)	12.3 (11.8–12.8)	18.7 (18.3–19.2)
Packaging and labeling material and equipment	9.1 (8.5–9.8)	3.2 (2.7–3.9)	17.5 (15.8–19.4)	22.7 (21.4–24.0)	14.7 (13.9–15.6)	11.6 (11.1–12.1)
Sharp instruments	96.6 (96.5–96.7)	91.2 (90.6–91.8)	94.8 (93.8–95.6)	96.7 (95.2–97.8)	98.5 (98.0–98.9)	94.6 (94.1–95.0)

^a^ positive aspects

^b^ negative aspects

* p < 0.05

Source: PNAUM – Services, 2015.

The little care with the measurement of temperature and humidity stands out, especially in hotter and more humid regions, such as the North and Northeast. In the observation, 3.5% in Brazil and 5.8% in the Northeast showed temperatures above 30°C. We observed locations with incidence of direct sunlight on medicines, signs of the presence of rodents and insects, and mold or infiltrations in the walls in all regions, conditions that can affect the stability of medicines.

In the dispensing areas, the environmental conditions were also poor, varying between the regions, with statistically significant differences in most items. In these areas, they were slightly more favorable than in the storage areas regarding temperature and humidity control and internal air circulation system; however, they were more unfavorable for allowing direct sunlight incidence on medicines, signs of the presence of insects and rodents, and mold or infiltrations in the walls.

The conditions of dose fractioning of medicines were also unfavorable and varied between the regions, with statistically significant differences: from the 711 observed units that fractionated medicines, only 12.8% had specific area, 18.7% had countertop coated with smooth and resistant material, and 11.6% had packaging and labeling material and equipment. Thus, we verified the noncompliance with fundamental requirements on the handling of medicines, with infringement of the health legislation.

As the Figure shows, the problems related to the transportation of medicines are distributed unevenly between the regions: the most frequent situation is that of insufficient vehicles, mainly in the Northeast and North regions. Insufficiency leads to the use of unsuitable vehicles, a situation that prevailed in the North region. South and Northeast were the ones that most accumulated the situation of insufficient and inadequate vehicles, ranking second in Brazil.

**Figure:**
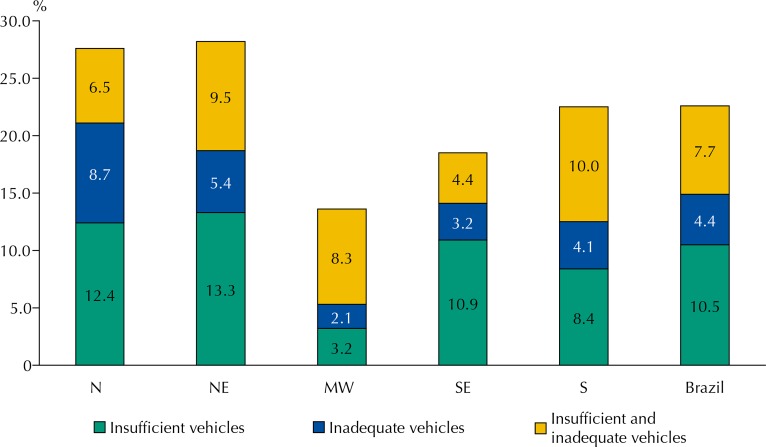
Problems of medicine transportation in Primary Health Care, in the regions of Brazil, according to the professional responsible for the municipal pharmaceutical services. National Survey on Access, Use and Promotion of Rational Use of Medicines – Services, 2015.

N: North; NE: Northeast; MW: Middle-West; SE: Southeast; S: South.

Source: PNAUM – Services, 2015.

As Table 3 shows, the existence of the norm to regulate the activities of advertising/promotion of medicines – access to the services of representatives of laboratories and distributors of medicines, distribution of advertising materials in the public health network, and distribution of free sample – varied between the regions, with statistically significant differences in the norm item. The South and Southeast regions had the highest percentages of cities that govern such practices and, in Brazil as a whole, the percentage reached 24.2%. However, 27.5% of respondents could not comment on this issue.

**Table 3:** Regulation of the advertising of medicines, pharmacovigilance, and waste management in the Primary Health Care, in the regions of Brazil, according to pharmacists responsible for dispensing of medicines. National Survey on Access, Use and Promotion of Rational Use of Medicines – Services, 2015. (n = 285)

Dimension/Variable	North	Northeast	Midwest	Southeast	South	Brazil
					
	% (95%CI)	% (95%CI)	% (95%CI)	% (95%CI)	% (95%CI)	% (95%CI)
Regulation of advertising/promotion of medicines						
Norm to regulate visits from representatives of laboratories and distributors of medicines and advertising material for medicines*	21.0 (12.9–32.2)	13.1 (7.6–21.5)	20.6 (11.9–33.1)	37.7 (22.8–55.3)	25.8 (18.5–34.8)	24.2 (18.1–31.5)
Does not know	27.2 (18.0–39.0)	31.4 (21.9–42.7)	20.1 (11.4–32.9)	25.6 (15.1–40.0)	25.3 (16.9–36.2)	27.5 (21.9–33.9)
Distribution of free samples of medicines in the health unit	33.6 (26.6–41.4)	37.3 (26.8–49.3)	26.9 (17.9–38.4)	26.0 (13.4–44.4)	37.4 (25.9–50.4)	32.8 (26.2–40.2)
Does not know	1.5 (0.6–3.7)	0.9 (0.2–3.7)	9.2 (1.8–36.0)	2.7 (0.7–9.4)	3.5 (0.9–12.9)	2.4 (1.2–4.8)
Pharmacovigilance*						
Mechanism for notification of technical complaints and adverse events caused by medicines	23.3 (15.9–32.9)	20.0 (14.8–26.5)	31.4 (17.9–48.9)	49.8 (36.0–63.6)	25.6 (17.3–36.1)	31.4 (25.3–38.2)
Has notified technical complaint or adverse event caused by medicines	3.9 (2.0–7.4)	4.7 (2.3–9.4)	13.5 (4.3–35.0)	24.6 (13.1–41.4)	9.9 (4.7–19.6)	12.3 (7.9–18.8)
Referral of technical complaints and notifications of adverse events caused by medicines						
To the pharmaceutical supply center	8.1 (3.9–16.4)	7.3 (4.1–12.6)	0.3 (0.1–1.0)	10.8 (2.7–34.7)	2.6 (1.4–4.7)	7.3 (3.7–14.0)
To the municipal coordination of pharmaceutical services	20.0 (14.5–27.0)	11.0 (6.6–17.8)	36.1 (22.4–52.7)	28.5 (14.6–48.2)	18.7 (8.3–36.9)	20.1 (14.2–27.7)
To the sanitary surveillance department	17.8 (8.1–34.8)	16.4 (8.2–30.0)	18.7 (10.3–31.3)	19.0 (10.6–31.7)	24.1 (14.7–37.0)	18.8 (13.8–25.1)
None	16.5 (10.2–25.6)	25.3 (13.3–42.8)	5.8 (2.9–11.3)	10.6 (5.7–18.9)	19.2 (11.8–29.8)	17.7 (10.2–25.4)
Other referrals	36.0 (26.8–46.4)	39.9 (27.8–53.3)	38.9 (26.0–53.5)	31.1 (17.0–49.9)	34.2 (23.3–47,1)	35.7 (28.6–43.5)
Inventory control system*						
Manual	83.9 (75.5–89.7)	87.8 (78.5–93.4)	39.0 (25.4–54.5)	36.4 (20.1–56.5)	28.4 (19.6–39.3)	58.2 (49.7–66.2)
Computerized	12.0 (7.1–19.6)	10.3 (4.8–20.7)	60.2 (44.4–74.1)	54.3 (36.1–71.5)	66.2 (54.7–76.1)	36.8 (29.4–45.0)
Does not have	4.1 (2.2–7.6)	1.9 (0.8–4.8)	0.8 (0.1–4.4)	9.3 (1.9–34.8)	4.9 (1.5–14.8)	4.9 (1.8–12.4)
Waste management of medicines						
The health unit has Waste Management Plan*	25.7 (18.4–34.8)	31.2 (20.0–45.1)	54.2 (41.3–66.6)	53.7 (36.8–69.8)	57.4 (44.2–69.7)	43.7 (35.9–51.7)
Specific and suitable location for storage of medicines in accordance with the norms*	23.9 (14.0–37.7)	22.6 (14.2–34.0)	37.5 (24.7–52.4)	65.0 (51.6–76.4)	39.3 (27.1–52.9)	39.9 (32.6–47.7)
Specific location for storage of waste of medicines noncompliant with the norms*	20.3 (14.6–27.5)	17.0 (11.3–24.9)	22.7 (12.9–36.8)	15.0 (8.6–24.9)	34.3 (21.9–49.3)	19.9 (15.7–25.0)
There is no specific location	54.6 (42.4–66.3)	51.4 (40.5–62.3)	34.2 (24.2–45.9)	17.4 (9.1–30.7)	23.7 (14.2–37.0)	35.2 (28.9–42.0)
Collection service of waste of medicines from the pharmacy/dispensing unit*	80.8 (69.1–88.8)	66.0 (53.6–76.5)	89.1 (79.3–94.6)	93.1 (86.9–96.5)	84.0 (68.2–92.8)	80.3 (74.7–84.9)

* p < 0.05

Source: PNAUM – Services, 2015.

Regarding the distribution of free samples, in Brazil, this practice was referred to exist in about 33% of health units. The Midwest showed the highest percentage of pharmacists who claimed not to know information about the topic, and the distribution of free sample reached nearly 27% of the units. It was not possible to investigate whether the norms of the cities addressed the distribution of free samples.

The initiatives regarding pharmacovigilance are distributed unevenly between the regions, with statistically significant differences. The percentage of units that had some mechanism for notification of technical complaint and/or adverse events related to medicines reached only 31.4% and the most favorable region was the Southeast, with 49.8%. We observed a disconnection between mechanisms and notification: when questioned if they had already carried out a notification, the percentage of positive answers from pharmacists are much lower than those relating to the existence of mechanisms for notification; this indicates that, in general, the services are establishing mechanisms for the notifications, but pharmacists are not yet accompanying the initiatives, mainly in the North and Northeast regions (3.9% and 4.7%, respectively).

The notifications have several referrals and vary between the regions: in Midwest, Southeast, and North they go mainly to the coordination of pharmaceutical services, while in the South and Northeast regions they go more to the Health Surveillance Department, referring also to the use of the *Sistema de Notificações em Vigilância Sanitária* (NOTIVISA – Notification System in Health Surveillance) of Anvisa. However, in Brazil, 17.7% of respondents stated that they do not give any referral to the notifications. This situation, along with the various procedures, indicate the early stage of organization of pharmacovigilance activities in the Primary Health Care; notifications are also sent to the *Central de Abastecimento Farmacêutico* (CAF – Pharmaceutical Supply Center), municipal secretariat of health, regional health board, ombudsman office, among others.

The existence of inventory control systems also showed inequalities between the regions, with statistically significant differences and predominance of manual control (58%), while about 5% of pharmacies/dispensing units do not have any control system.

The existence of *Plano de Gerenciamento de Resíduos de Serviços de Saúde* (PGRSS – Health Care Services Waste Management Plan) varied between the regions, with statistically significant differences in most items in this dimension: North and Northeast are the poorest regions. In Brazil, less than 44% of health units comply with this legal requirement. Regarding the specific location to store medicines unfit for use until they are collected, only 40% of the units in Brazil have suitable place, and respondents reported, at high percentages, only one waste collection service, but it was not possible to investigate the local of deposition. The disposal of medicines was the subject of a study in the context of Family Health, which found little understanding from workers about appropriate disposal, execution of practices different from legal devices, and disarticulation between sanitary surveillance and other health services^1^.

## DISCUSSION

The findings of this study in many aspects corroborated with studies on pharmaceutical services (PS) in Brazilian Primary Health Care. In a study regarding the professional responsible for pharmacy/dispensing unit, observed that, in Brasília (Federal District), of the 15 units studied, only two had pharmacist as responsible^20^, a technical and a legal requirement. In Rio Grande do Sul, in 20 cities of the 17^th^ Regional Health Coordination, only five had pharmacists^8^. Barreto and Guimarães^3^ also found, in cities from Bahia, the absence of pharmacist in carrying out essential activities, such as scheduling and dispensing of medicines.

The dimensions investigated showed results that indicate sanitary conditions inadequate to medicines and reveal that the sanitary surveillance norms and the recommendations of the Brazilian Ministry of Health to guide the structuring of pharmacies of SUS are not yet much followed. It should be stressed that the storage of medicines involves a number of technical and administrative procedures; when suitable, the storage reduces the loss of medicines and is essential for the preservation of their quality^d^.

The findings about the environmental and storage conditions are worrisome, especially considering the weather conditions in the Country, with high temperatures in most of the territory and throughout the year and with a lot of moisture. It should be stressed that the quality and efficacy of the medicine is directly related to the maintenance of its stability regarding storage conditions and handling^13^, including during fractioning. Poorly preserved medicines carry risks to the patient’s health, risks associated with the reduction or absence of the therapeutic effect, and adverse events resulting from changes in the pharmaceutical formulation, from heat, humidity, and/or decomposition caused by ultraviolet rays^14^. In the case of malaria, e.g., the use of poor quality and/or false medicines can contribute to develop the resistance of the parasite^21^.

The deficient conditions we found confirm the findings of other studies: from data collected from the monitoring reports of cities, prepared by the Comptroller General of Brazil, Vieira^26^ found problems in management or services in 90.3% of the 597 cities supervised, which are 10.7% of the Brazilian cities. In addition to inadequate storage conditions in 39% of the cities, the study showed that these inadequacies represent the second leading cause of lack of medicines, found in 24% of the cities. Barreto and Guimarães^3^ also found the absence of physical and environmental conditions ideal for the storage of medicines and inadequate temperature in these locations, both in the CAFs and in the *Unidades Básicas de Saúde* (UBS – Basic Health Units).

In an evaluation study of pharmaceutical services in Brazil^22^, the storage conditions in the health units were adopted as a proxy for the quality of medicines. On a scale from zero to 100 points, half the units reached between 40 and 69 points regarding good storage practices in CAF, which may indicate that the medicines could be subjected to inadequate conditions in some aspects. The conditions were considered reasonable, with wide variation between the services inspected.

These conditions can be exacerbated by the way medicines are transported. Inadequate transport might compromise the quality and efficacy of medicines, by exposing the products to high temperatures, poor cleaning condition of vehicles, handling, and others factors^14^. Barreto and Guimarães^3^ also found an inadequate transport situation in the cities studied.

Regarding the existence of inventory control systems, the results of this study, despite indicating the need for better quality control, were more favorable than in other studies that evaluated pharmaceutical services in Brazil and found records of inventory in 32% of health units, 32% in the municipal CAF, and 61% in state stations, revealing the precariousness of inventory control in the services^22^. Vieira^26^ found the lack or deficiency in this control in 71% of the sample of cities in his study. Barreto and Guimarães^3^ also found the lack of inventory control in the cities studied, which may affect the availability of medicines.

While investigating the existence of norms to regulate the activities of advertising/promotion of medicines in the services, almost 28% of pharmacists could not comment on the issue; however, the distribution of free samples was reported in about a third of the health units in Brazil. It should be stressed that the industries invest heavily in strategies of medicine promotion, with visits of representatives who usually provide free samples as a way of marketing the pharmaceutical laboratory^13^. The regulation of advertising/promotion of medicines in cities and states is a necessity of the health system. As shown by the studies, the advertisement can change the prescription pattern of physicians^4^ ^,^ ^9^ ^,^ ^12^ ^,^ ^19^ ^,^ ^23^ ^,^ ^25^.

In the study by Fagundes et al.^9^ with a group of 50 physicians, 98% reported receiving regular visits from sales representatives of the pharmaceutical industry: 40% claimed to receive them weekly, and 12% received daily. Almost half (45%) claimed to have visitors from different propagandists publicizing the same product and 86% reported receiving gifts in the visits. Among the physicians, 68% believed there was direct influence of propaganda on the prescriptions; however, most of them claimed not to be influenced by the propaganda and 14% said they prescribe medicines because of the receipt of prizes. Regarding the advertisements, 22% said to rely fully on the information received and 68% believed there are untruths or inaccuracies.

Despite Anvisa regulations, many issues related to the distribution of free samples require discussion and regulation^11^: the definition of the quantity delivered to each prescriber, the minimum period allowed for their distribution, transportation mechanisms, storage and distribution control, and care with the expiration date. Authors^24^ confirm these evaluations, acknowledging that there have been advances, but they consider the standardization still incipient and postulate the need for discussions following technical-scientific criteria with the least possible influence of the pharmaceutical industry, for greater safety and effectiveness in the use of free samples of medicines.

Regarding the initiatives on pharmacovigilance, the findings indicate an early stage of organizing these activities in the primary health care, with statistically significant differences between the regions. Brazil is laggard in the development of this practice, which has the important function of contributing to the sanitary regulation on improving the safety profile of medicines. After the creation of Anvisa, a process for the institutionalization of pharmacovigilance began, which is still not a relevant practice in health services, except in hospitals from the *Rede Sentinela* (Sentinel Network)^16^.

The sanitary conditions found in pharmacies/medicine dispensing units in the primary health care are unfavorable and alarming to the conservation of medicines. Medicines are delicate and sensitive products, manufactured in strict technical basis; they require specific conditions of transportation, storage, and handling and also the surveillance of adverse events, besides the control of their advertising/promotion, as part of the sanitary regulatory system. It should be stressed that the logistics cycle of pharmaceutical services, based on the foundation of rational use of medicines, cannot do without the pharmacist, who is also instrumental in the care of the user of these technologies. These items, among others, show profound disabilities.

Although it was not possible to investigate the conditioning factors of the reality found, we observed that, in general, the sanitary situation in pharmaceutical services in the primary health care/SUS faces the noncompliance with items indispensable for the exercise of activities with these health inputs. The results indicate that health services face management problems that reveal precarious infrastructure, organization, and quality of pharmaceutical services, which can have a negative impact on the quality, efficacy, and safety of the medicines, in addition to the costs. A limitation of this study is that no comprehensive studies were found, which would allow comparisons; however, the indicators identified provide subsidies for the improvement of health policies in this particular component. The findings allow to conclude that advances were observed, but they are still timid, notably in the Northeast and North regions; and that there is urgency for management responsibility and organization and qualification of pharmaceutical services in the primary health care, so that PS and drug policies meet their purpose in SUS.

## Footnotes

a Portaria 3916 do Ministério da Saúde, de 30.10.1998, aprova a Política Nacional de Medicamentos. Available from: http://bvsms.saude.gov.br/bvs/saudelegis/gm/1998/prt3916_30_10_1998.html

b Ministério da Saúde (BR), Agência Nacional de Vigilância Sanitária. Resolução RDC nº 306, de 7 de dezembro de 2004. Dispõe sobre o Regulamento Técnico para o gerenciamento de resíduos de serviços de saúde. Diario Oficial Uniao. 10 dez 2004; Seção 1:49.

c Ministério do Meio Ambiente(BR), Conselho Nacional do Meio Ambiente - CONAMA. Resolução nº 358, de 29 de abril de 2005. Dispõe sobre o tratamento e a disposição final dos resíduos dos serviços de saúde e dá outras providências. Diario Oficial Uniao. 4 maio 2005; Seção 1:63-5.

d Conselho Nacional de Secretários de Saúde. Assistência Farmacêutica no SUS. Brasília (DF): CONASS; 2011. (Coleção Para Entender a Gestão do SUS, 7).
